# Growth differentiation factor 15 contributes to marrow adipocyte remodeling in response to the growth of leukemic cells

**DOI:** 10.1186/s13046-018-0738-y

**Published:** 2018-03-22

**Authors:** Wei Lu, Yun Wan, Zhiqiang Li, Bin Zhu, Chunrong Yin, Haiyan Liu, Shaoxin Yang, Yuanmei Zhai, Yehua Yu, Yanyu Wei, Jun Shi

**Affiliations:** 1 Department of Hematology, Shanghai Jiao Tong University Affiliated Sixth People’s Hospital, Shanghai, China; 2 Department of Blood Tranfusion, Shanghai Jiao Tong University Affiliated Sixth People’s Hospital, Shanghai, China; 3 Department of Hematology, Shanghai Jiao Tong University Affiliated Sixth People’s Hospital South Campus, Shanghai, China; 4 Department of Hematology, Tongren Hospital Shanghai Jiao Tong University School of Medicine, Shanghai, China; 5 Department of Hematology, Shanghai Jiao Tong University School of Medicine Affiliated Ninth People’s Hospital, Shanghai, China

**Keywords:** Acute myeloid leukemia, GDF15, Marrow adipocyte, Adipocyte remodeling

## Abstract

**Background:**

The adipocyte remodeling, including of the morphological change, might indicate special pathological function. Our previous study found that the morphological remodeling of larger marrow adipocytes into small marrow adipocytes correlates with a poor prognosis for acute myeloid leukemia (AML) patients. However, the mechanisms contributed to the marrow adipocyte remodeling are still poorly understood.

**Methods:**

GDF15 expression was analyzed by RT-qPCR and western blotting assays in the leukemic cells. The enhancing and antibody neutralization tests in vitro were employed to evaluate the effect of GDF15 on the morphology of mature adipocytes. CCK8 test was used to detect the proliferation of leukemic cells after co-cultivation with small marrow adipocytes. Flow cytometry was used to analysis the proportion of cell cycle of leukemic cells. Immunofluorescence staining and linear analysis were applied to verify the GDF15 expression and the relationship between GDF15 and small marrow adipocytes in AML patients.

**Results:**

In this study, we found that leukemic cell lines not only expressed significantly higher growth differentiation factor 15 (GDF15) than the other three cytokines associated with adipocyte differentiation in RNA level but also secreted GDF15 factor. Furthermore, the in vitro experiments demonstrated that GDF15 was involved in the conversion of small marrow adipocytes from larger marrow adipocytes. Correspondingly, the leukemic cells proliferated more rapidly through regulating the cell cycle when co-cultured with GDF15-induced small marrow adipocytes. The immunofluorescence staining on the bone marrow sections of AML patients further exhibited that GDF15 was partly produced by leukemic cells. The positive correlation between the concentration of GDF15 in the marrow aspirates and the number and the volume of small marrow adipocytes might suggest the contribution of GDF15 in AML patients (*r* = 0.72, *r* = 0.67).

**Conclusions:**

GDF15 secreted by leukemic cells was involved in the morphological remodeling of marrow adipocytes, which can in turn promote leukemic cell growth, indicating that GDF15 may be a promising treatment target for AML patients.

**Electronic supplementary material:**

The online version of this article (10.1186/s13046-018-0738-y) contains supplementary material, which is available to authorized users.

## Background

Acute myeloid leukemia (AML) is a malignant hematological disease that occurs primarily in the bone marrow (BM). Marrow adipocytes, as an important component of the BM niche, have been suggested to contribute to the proliferation and anti-chemotherapy of AML cells by providing energy or secreting adipokines [1, 2]. Our previous study found that only small marrow adipocytes, not the total marrow adipocytes, were correlated with a poor prognosis for AML patients [3], suggesting that understanding the generating mechanism of small marrow adipocytes may be useful for improving the prognosis of AML patients.

In prostate cancer and ovarian cancer, it has been reported that adipocytes surrounding the tumor cells become small and are involved in the metastasis and growth of prostate cancers or ovarian cancers [4, 5]. In leukemia, Shafat et al. reported that marrow adipocytes transfer fatty acids to AML blasts by activating lipolysis in adipocytes [2]. These reports indicate that the reduction of adipocyte size is due to transfer of their lipid droplets to tumor cells. Additionally, in breast cancer, tumor cells release inflammatory factors, such as TNFα and Wnt3a, which are involved in the regulation of morphological remodeling of the mature adipocytes, including the reduction of adipocyte size and acquisition of fibroblast-like morphology [6]. This implies that the morphological remodeling of adipocytes are not only due to the transfer of their lipid droplets to the surrounding tumor cells but also highly dependent on extrinsic signals from tumor cells.

Growth differentiation factor-15 (GDF15), a TGF-β/bone morphogenetic protein (BMP) superfamily member, is a 40-kDa secretory propeptide that is cleaved in the endoplasmic reticulum to release a 25-kDa circulating protein [7]. Under physiological conditions, GDF15 is abundantly expressed only in placenta and macrophage cells [8]. However, recent studies have reported that GDF15 is highly expressed in many types of cancer tissues, including colorectal, gastric, esophageal, oral, pancreatic and so on [9–14]. We previously found the expression of GDF15 was high in the residual acute lymphoblastic leukemic cells in mice [15]. However, its expression in AML cells has not been studied in depth. Studies have shown that similar to other TGF-β family members, GDF15 is involved in the inhibition of cell growth, induction of apoptosis, and enhancement of cancer invasiveness in different cancer cell lines [16–19]. Until now, little attention was paid to the effect of GDF15 on the adipocytes. Increased serum concentrations of GDF15 have been reported in patients with anorexia nervosa and obesity [20], inspiring us to explore the function of GDF15 on the adipocytes. Here, we demonstrate that leukemic cells highly express GDF15, and in turn, GDF15-induced small adipocytes may promote the growth of leukemic cells.

## Methods

### Cell culture and regents

The leukemic cell lines THP-1, K562, HEL, HL-60 and Kasumi (Chinese Academy of Sciences Cell Bank, Shanghai, China) were cultured in 1640 supplemented with 10% fetal bovine serum (FBS) (Gibco, Grand Island, NY, USA) and penicillin-streptomycin at 37 °C in 5% CO2. Primary AML blasts and mesenchymal stem cells in bone marrow (BMSCs) were isolated by Ficoll-Hypaque (Axis-Shield Diagnostics, Dundee, Scotland, UK) density-gradient centrifugation. BMSCs were differentiated into adipocytes as previously described [21]. The differentiated adipocytes were stained with Oil Red O (ORO). Conditioned medium (CM) from the leukemic cell lines was obtained from cells cultured with high glucose Dulbecco’s modified Eagle’s medium (DMEM) supplemented with 1% FBS. Mature adipocytes were cultured with high glucose DMEM supplemented with 10% FBS alone or mixed with the leukemic cell lines CM at the ratio of 4:1. Mature adipocytes were treated with recombinant human GDF15 (rhGDF15 200 ng/ml, Peprotech, Cat#120–28, USA) or a neutralizing anti-GDF15 antibody (8 μg/ml, R&D Systems, Cat#MAB957, USA) for 5 days to observe the effect of GDF15 factor on the marrow adipocytes.

### Cell cycle analysis

The measurements were made using a flow cytometry (Beckman, Urbana, IL, USA). In brief, THP-1 and K562 cells were cultured with the CM from mature adipocytes and small adipocytes for 48 h respectively, washed with PBS, then fixed with 70% ethanol for 24 h. Cells were incubated with propyl iodide (Sigma, St Louis, MO, USA) organism dye for 30 min at 37 °C, followed by flow cytometric analysis.

### Free fat acid detection

Adipocytes were treated with or without rhGDF15 (200 ng/ml) for 5 days, then the cultured medium was replaced by high glucose DMEM with 1% FBS for 48 h. The supernatant of adipocytes was collected and stored in − 80 °C.The concentration of FFA was detected by using a colorimetric method via a commercial kit (Sigma Aldrich, St. Louis, Missouri). The assay was carried out according to manufacturer’s specifications.

### Cell proliferation assay

After treating mature adipocytes with GDF15 for 5 days, we cultured the GDF15-induced small adipocytes with DMEM supplemented with 10% FBS for 48 h. Then, leukemic cells were co-cultured with the CM of mature adipocytes or GDF15-induced small adipocytes. Leukemic cells (3 × 10^3^) were seeded into 96-well plates, and the leukemic cell proliferation was evaluated by a CCK8 kit (Dojindo, Japan). CCK8 reagent was added to each well and incubated for 2 h at 37 °C. The measurement of absorption at 450 nm was performed using a microplate reader (Multiscan FC, Thermo Fisher, USA).

### Transfection assays

Transfection assays were set up for 4 groups, scrambled siRNA (negative control/NC), GDF15-homo-161 (siRNA-161); GDF15-homo-290 (siRNA-290) and GDF15-homo-860 (siRNA-860) groups. GDF15-siRNA and scrambled siRNA were obtained from Shanghai GenePharma Co., Ltd (Shanghai, China) and blended into 20 μM with DEPC water and stored in - 20 °C. The THP-1 cells were seeded in 24-well plates at a density of 2 × 10^5^ cells/well for 24 h prior to transfection and then the medium was replaced with 0.5 mL of Opti-MEM medium. Subsequently, 1 μL of Lipofectamine 2000, 2 μL of GDF15-siRNA was added into the corresponding well. After 48 h and 72 h of transfection, the cells were collected for further gene expression assays.

### PCR and real-time quantitative PCR (RT-qPCR)

Total RNA was extracted using Trizol (Invitrogen, Paisley, UK), and the RNA was converted into cDNA using the PrimeScript™ RT reagent Kit (Takara Bio Inc, Shiga, Japan) for RT-qPCR and using the PrimeScript™ II 1st Strand cDNA Synthesis Kit (Takara Bio Inc, Shiga, Japan) for PCR. All RT-qPCR reactions were performed using an ABI 7500 system (Biosystems, Foster City, CA, USA) and the SYBR Premix Ex Taq reagent kit (Takara Bio Inc, Shiga, Japan). Premix Taq™ (Takara Bio Inc, Shiga, Japan) for electrophoresis. All the primers used in this study are presented in the Table 1.

**Table 1:** Sequences of the primers used to detect genes expression by RT-qPCR

Name	Forward	Reverse	GeneBank Accession
GDF15	GACCCTCAGAGTTGCACTCC	GCCTGGTTAGCAGGTCCTC	NM_004864
FABP4	AACCTTAGATGGGGGTGTCCTG	CTCTCTCATAAACTCTCGTG	NM_001442
PPARγ	GGGATCAGCTCCGTGGATCT	TGCACTTTGGTACTCTTGAAGTT	NM_138711
C/EBPα	GAACAGCAACGAGTACCGGGTA	GCCATGGCCTTGACCAAGGAG	NM_004364
HSL	GACCCCTGCACAACATGATG	TGAGCAGCACCCTTTGGATG	NM_005357
ATGL	GGCTTCCTCGGCGTCTACTA	TTTACCAGGTTGAAGGAGGGG	NM_020376
GAPDH	AATGGACAACTGGTCGTGGAC	CCCTCCAGGGGATCTGTTTG	NM_014364

### Western blotting analysis and ELISA

Mononuclear cells from the controls and primary AML patients were washed twice with phosphate buffer saline and resuspended in 200 μl of lysis buffer containing a mix of protease inhibitors (Beyotime, Haimen, Jiangsu, China). Immunoblots were prepared as previously described [22]. Rabbit anti-GDF15 monoclonal antibody (Abcam, Cambridge, MA, USA), rabbit anti-β-tubulin antibody (Abcam, Cambridge, MA, USA), rabbit anti-Cdk2 antibody (Abcam, Cambridge, MA, USA), rabbit anti-Cyclin D1 antibody (Abcam, Cambridge, MA, USA), rabbit anti-P21 antibody (Abcam, Cambridge, MA, USA) and rabbit anti-β-actin monoclonal antibody (Cell Signaling Technology, Danvers, MA, USA). The THP-1 cell CM was obtained from cells cultured in regular medium with 1% FBS at different cell densities (2 × 10^5^/ml, 5 × 10^5^/ml, 1 × 10^6^/ml, 2 × 10^6^/ml). The ELISA analysis for GDF15 was performed according to the manufacturer’s instructions (R&D systems).

### Immunofluorescence

Adipocytes differentiated on coverslips grown alone or co-cultivated with leukemic cells were fixed with 4% paraformaldehyde and blocked with goat serum. The process was carried out as previously described [23]. Cells were stained with neutral lipid specific BODIPY®493/503 (4, 4-difluoro-1,3,5,7,8-pentamethyl-4-bora-3a,4a-diaza-s-indacene) dye, tubulin and DAPI. The immunofluorescence of BM paraffin sections from AML patients was performed as previously described [24]. The sections were stained with GDF15 (goat anti-GDF15 multiclonal antibody, Abcam, Cambridge, MA, USA), CD34 (rabbit anti-CD34 monoclonal antibody, Abcam, Cambridge, MA, USA), CD117 (rabbit anti-CD117 polyclonal antibody, Proteintech, Wuhan, China) and DAPI (Solarbio, Beijing, China). Fluorescent images were captured using a confocal laser microscopy system (Leica SP2).

### Adipocyte measurements

BM trephine biopsies of 20 AML patients were obtained from the posterior iliac crest, and BM tissues were fixed, decalcified and embedded with paraffin or plastic slices according to the conventional methods. This study was approved by the Medical Ethical Committee of our institute. The adipocyte number and adipocyte volume were measured as previously reported. The number and area of per adipocyte cultured in vitro were measured by using Image-Pro Plus 5.1. Ten fields were analyzed at × 400 magnification.

### Statistical analysis

Values were calculated as the mean ± SEM. The Spearman’s correlation test was used to analyze the correlation between small adipocyte volume or small adipocyte number and GDF15 level in the BM. For all analyses, *P* < 0.05 was considered to be significant. All statistical analyses were performed using the SPSS 20.0 software program (Statistical Package for Social Science, SPSS Inc. Chicago, IL., USA).

## Results

### The soluble cytokines secreted by leukemic cells contribute to adipocyte remodeling

**Fig. 1:**
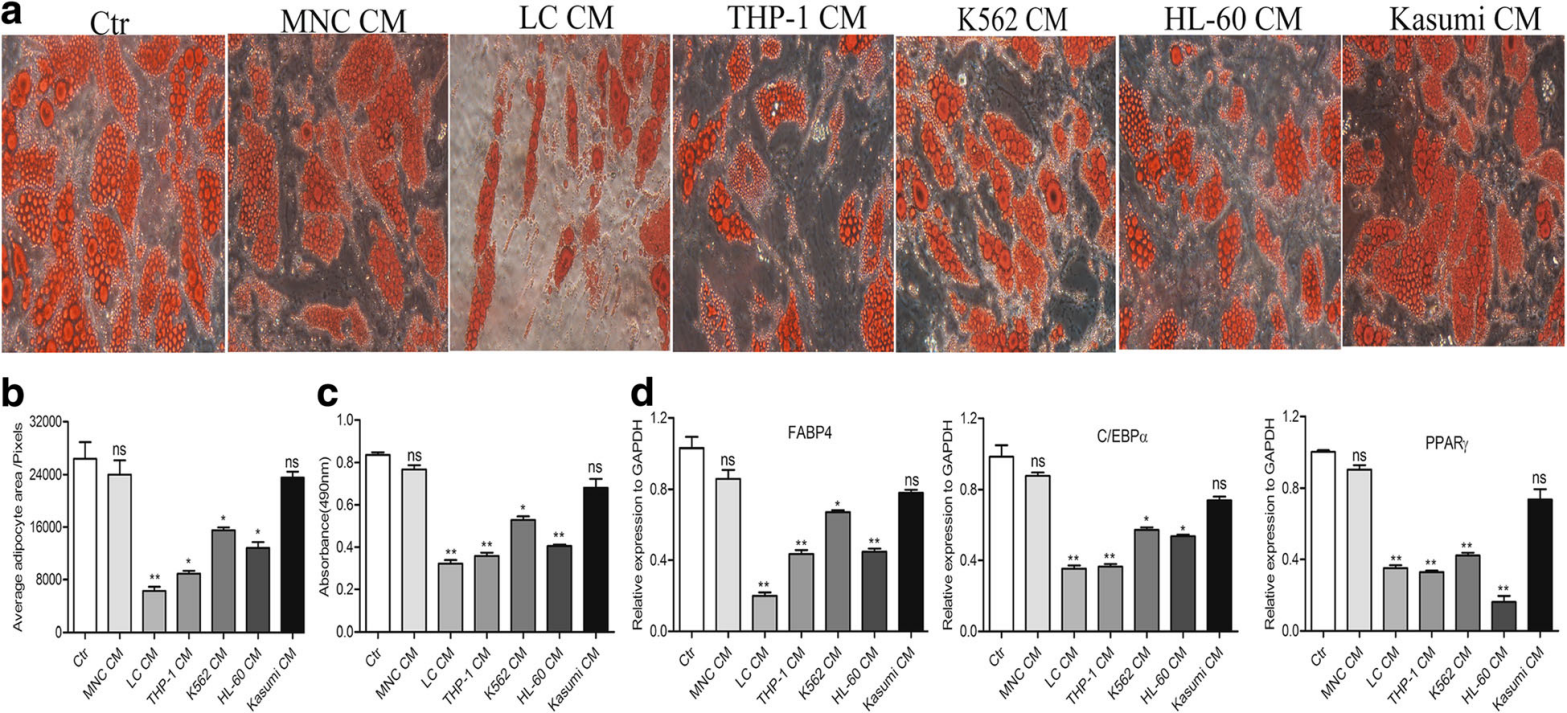
The soluble cytokines secreted by leukemic cells contribute to the adipocyte remodeling. **a** BMSC-derived adipocytes cultured with HG-DMEM supplied with 10% FBS (Ctr) or with the conditioned medium (CM) of healthy mononuclear cells (MNC CM), primary leukemic cells (LC CM), THP-1 cell line (THP-1 CM), K562 cell line (K562 CM), HL-60 cell line (HL-60 CM) and Kasumi cell line (Kasumi CM) for 5 days. Adipocytes were stained by ORO. Both images are at a magnification of 400×. **b** The average area of adipocytes cultured with the CM of different AML cells were compared with the controls by using Image-Pro-Plus 5.1. *P < 0.05, **P < 0.01. **c** The content of adipocyte lipid-droplets in indicated groups was detected by OD values after ORO staining. *P < 0.05, **P < 0.01. **d** RT-qPCR analysis of adipogenic genes (*FABP4, C/EBPα* and *PPARγ*) in indicated groups. *P < 0.05, **P < 0.01. Results shown are from three independent experiments. Values shown are the mean ± SEM

Our previous study found that leukemic cells could induce the formation of small adipocytes in vitro [3]. To investigate the effect of soluble cytokines secreted by leukemic cells on BMSC-derived mature adipocytes, we cultured the mature adipocytes with the CM of different leukemic cells, including THP-1, K562, HL-60, Kasumi, primary AML blasts (LC) and healthy mononuclear cells (MNC) for 5 days. The ORO staining and the quantitative analysis of adipocyte area showed that except Kasumi cells, the average area of adipocytes cultured with the CM of different leukemic cell lines was significantly decreased (reduced by 48%~ 64%, *P* < 0.05, Fig. 1a-b) when compared with the control. Similar results were also observed when mature adipocytes cultured with LC CM, but not with MNC CM. Meanwhile, the dramatic reduction of the content of lipid-droplets in mature adipocytes was also observed by the detection of OD values and RT-qPCR analysis of adipogenic genes, including fatty acid binding protein (*FABP4*), peroxisome proliferator-activated receptor gamma (*PPARγ*) and CCAAT/enhancer binding protein alpha (*C/EBPα*) (Fig. 1c-d), suggesting that the soluble cytokines secreted by leukemic cells promote the remodeling of small adipocytes from larger adipocytes.

### GDF15 is highly expressed in leukemic cell lines

**Fig. 2:**
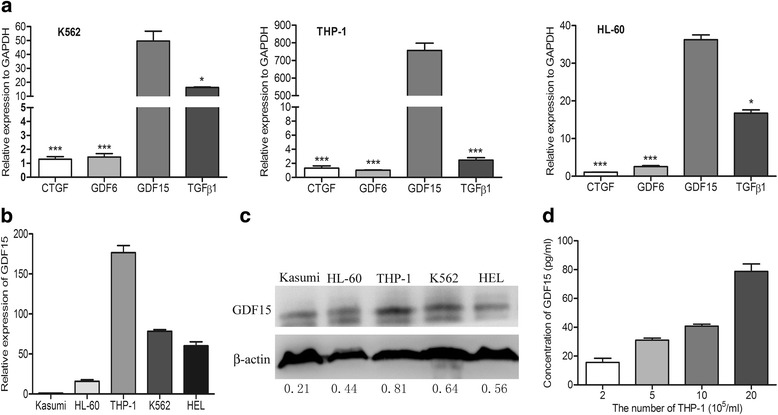
AML cell lines highly express GDF15. **a** RT-qPCR analysis of different cytokines associated with the regulation of adipogenesis in AML cell lines (K562, THP-1 and HL-60). *GAPDH* was used as a housekeeping gene. *P < 0.05, ***P < 0.001. **b** and **c**, RT-qPCR (**b**) and Western blotting (**c**) analysis of GDF15 in different cell lines (Kasumi, HL-60, THP-1, K562 and HEL). The densitometry values of protein expression changes were indicated. β-actin was used as an internal control for RT-qPCR and Western blotting analysis. **d** ELISA detection of GDF15 expression in the supernatant of THP-1 cells with different cell densities

To explore the critical cytokines secreted by leukemic cells, the mRNA levels of four cytokines that inhibited adipogenesis or promoted the transition to fibroblasts, such as TGF-β1, GDF15, growth differentiation factor 6 (GDF6) and connective tissue growth factor (CTGF) were detected by PCR and RT-qPCR assay. As shown in Fig. 2a, the expression of GDF15 was obviously higher than the other three factors in leukemic cells (*P* < 0.05 or *P* < 0.001). Furthermore, RT-qPCR and Western blotting analysis were performed to detect the GDF15 expression in different AML cell lines, including THP-1, K562, HEL, HL-60 and Kasumi. Our results demonstrated that THP-1 cells expressed the highest GDF15 among these cells (Fig. 2b-c); therefore, they were used in the following studies. ELISA assay showed that GDF15 factor was continuously released into the supernatant with the density of THP-1 cells increasing (Fig. 2d), indicating that AML cells can highly express and secrete GDF15.

### GDF15 is involved in the remodeling of larger adipocytes to small adipocytes

**Fig. 3:**
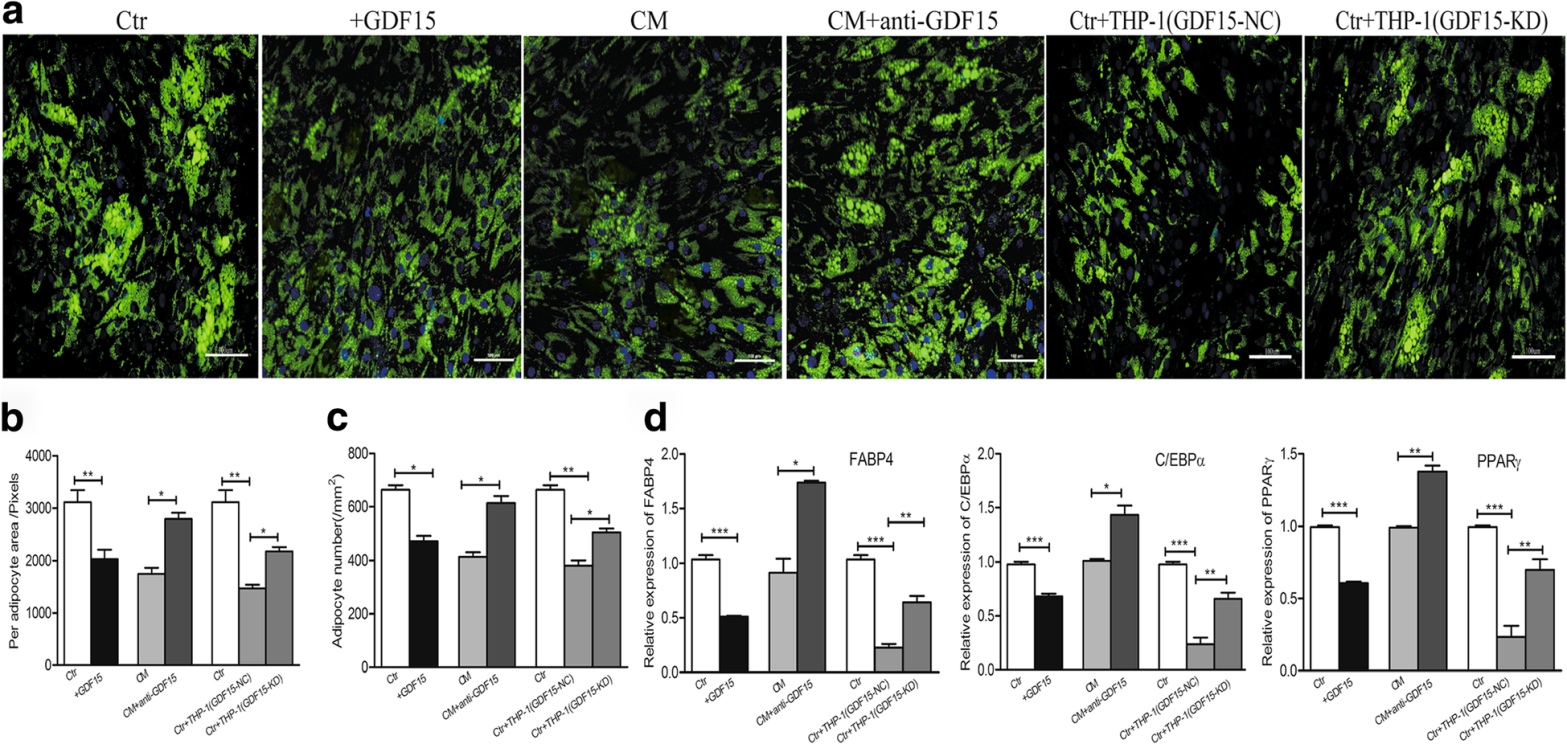
Contribution of GDF15 secreted by leukemic cells to the adipocyte remodeling. **a** Adipocytes treated with rhGDF15 (+GDF15) or neutralizing anti-GDF15 antibody (CM + anti-GDF15) or co-cultured with GDF15 knock down THP-1 cells (Ctr + THP-1 GDF15-KD) were stained with Alexa Fluor 493/503-conjugated BODIPY. Adipocytes cultured alone (Ctr) or cultured with the conditioned medium of THP-1 cells (CM) or co-cultured with negative control (Ctr + THP-1 GDF15-NC) were used as the controls respectively. Scale bar represents 100 μm. **b** The area of each adipocyte in each group was analyzed by using Image-Pro-Plus 5.1. *P < 0.05, **P < 0.01. **c** Adipocyte number in each group was analyzed by using Image-Pro-Plus 5.1. *P < 0.05, **P < 0.01. **d** The adipogenic gene (*FABP4, PPARγ, C/EBPα*) expression was detected by RT-qPCR analysis in each group. *P < 0.05, **P < 0.01, ***P < 0.001. Results shown are from three independent experiments. Values shown are the mean ± SEM

The biological effects of GDF15 on the adipocytes was further examined. After adding rhGDF15 into the culture medium of mature adipocytes for 5 days, adipocytes exhibited a decrease in the number and size of lipid droplets, as seen by the BODIPY staining (Fig. 3a), co-culturing mature adipocytes with the supernatant of THP-1 cells caused a reduction in the number and size of lipid droplets, while the addition of neutralizing anti-GDF15 antibody into the medium resulted in an increase in the number and size of lipid droplets (Fig. 3a). To further verify the function of GDF15 secreted by leukemic cells on mature adipocytes, we knocked down (KD) the GDF15 expression in THP-1 cells (Additional file 1: Figure S1) and assessed its effects on adipocytes. Adipocytes co-cultured with GDF15-KD THP-1 cells exhibited an increase in the number and size of lipid droplets when compared with adipocytes co-cultured with the negative control (NC) by the BODIPY staining (Fig. 3a). Subsequently, quantitative analysis showed a reduction in the area of each adipocyte and adipocyte number when adipocytes treated with rhGDF15 (Fig. 3b-c). However, an obvious increase in the area of each adipocyte and adipocyte number was observed when adipocytes were treated with GDF15 neutralizing antibody or co-cultured with GDF15-KD THP-1 cells (Fig. 3b-c). Additionally, we also observed that mature adipocytes exhibited a dramatic reduction in the adipogenic markers, including FABP4, PPARγ and C/EBPα by RT-qPCR analysis when they were treated with rhGDF15 (Fig. 3d). The expression of adipogenic markers increased when adipocytes treated with GDF15 neutralizing antibody or co-cultured with GDF15-KD THP-1 cells (Fig. 3d). These results suggested that it was GDF15 secreted by leukemic cells that promoted the remodeling of small adipocytes.

### GDF15-induced small adipocytes promote the growth of AML cells

**Fig. 4:**
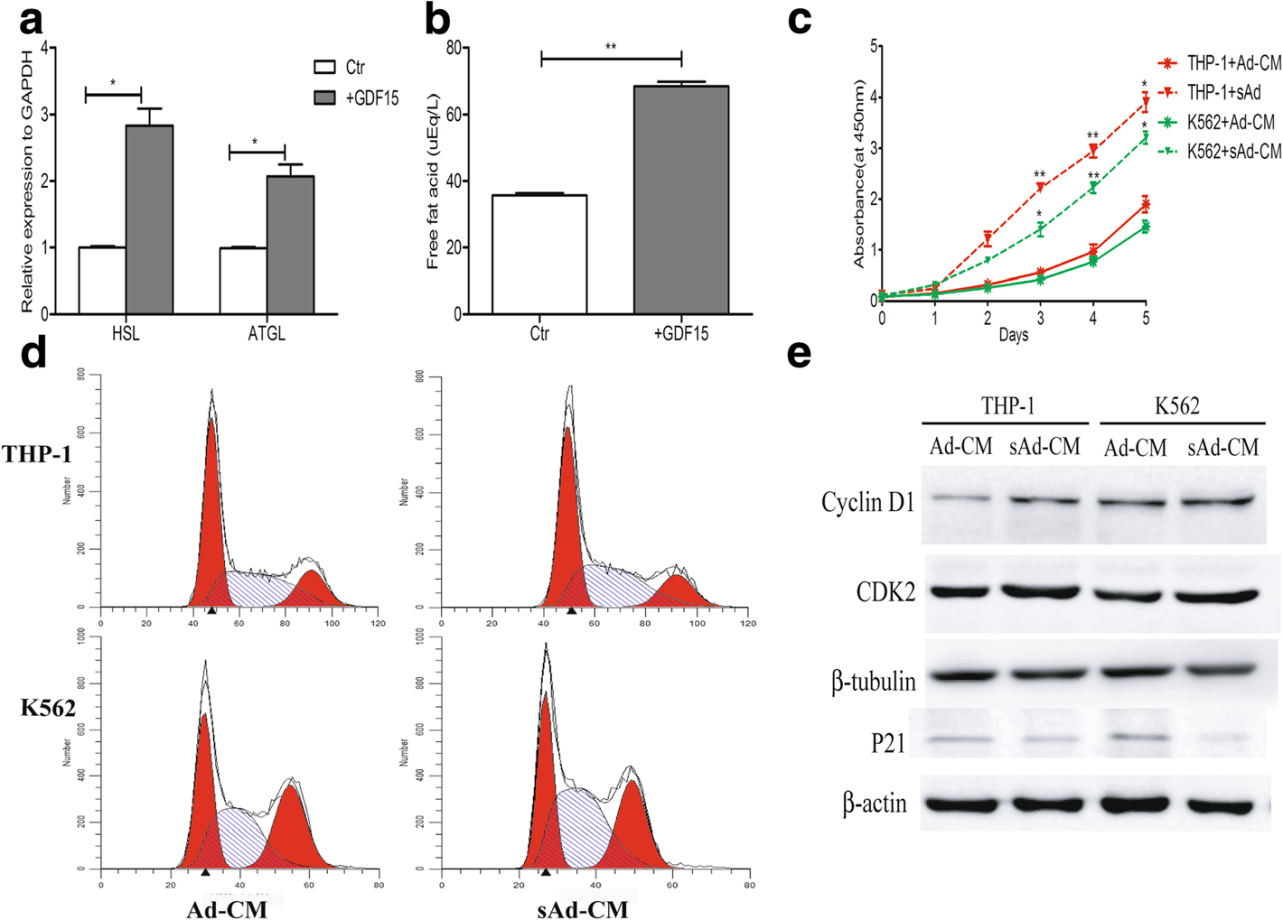
GDF15-induced small adipocytes promote the AML cells growth by increasing the lipolysis. **a** RT-qPCR analysis of lipolytic genes (*HSL* and *ATGL*) in adipocytes treated with rhGDF15 or cultured alone. *P < 0.05. **b** The content of free fat acid (FFA) in the supernatant of adipocytes from indicated groups was detected using the colorimetric method. **P < 0.01. **c** CCK8 detection of the proliferation of THP-1 cells and K562 cells cultivated with conditioned medium of adipocytes (Ad-CM) or small adipocytes (sAd-CM) from the indicated two groups. The statistical difference was compared between sAd-CM group and Ad-CM group of these two leukemic cells. *P < 0.05, **P < 0.01. Results shown are from three independent experiments. Values shown are the mean ± SEM. **d** Representative images showed the cell cycle of THP-1 cells and K562 cells treated with Ad-CM or sAd-CM. **e** Western blotting analysis of Cyclin D1, CDK2 and P21 protein levels in THP-1 and K562 cells treated with Ad-CM or sAd-CM. β-actin and GAPDH protein were used as internal controls for Western blotting analysis

Generally, the change of adipocyte morphology might indicate its abnormity in function [25–27]. We found that small adipocytes induced by GDF15 produced more free fatty acids (FFAs), resulting from the increase of lipolytic gene expression (hormone-sensitive triglyceride lipase, HSL and adipose triglyceride lipase, ATGL) (Fig. 4a-b). To investigate the effects of GDF15-induced small adipocytes on the proliferation of AML cells, we collected the CM from GDF15-induced small adipocytes and mature adipocytes. As shown in Fig. 4c, leukemic cell proliferation significantly increased when cultured with the CM from GDF15-induced small adipocytes compared with that from mature adipocytes, suggesting small adipocytes can promote the growth of leukemic cells. To study the mechanism of small adipocytes to enhance the leukemic cell proliferation, we analyzed the change of cell cycle of leukemic cells. The proportion of THP-1 cells and K562 cells in S phase was increased after treatment with the CM from small adipocytes when compared with that from mature adipocytes (40.03 ± 2.72% vs 33.63 ± 2.51%; 44.67 ± 2.31% vs 35.77 ± 2.07%, *P* < 0.05, respectively) (Fig. 4d). Furthermore, the protein expression of the cycle proteins CDK2 and Cyclin D1 were observed to increase while cyclin-dependent kinase inhibitor P21 decreased (Fig. 4e).

### GDF15 is associated with small adipocytes in AML patients

**Fig. 5:**
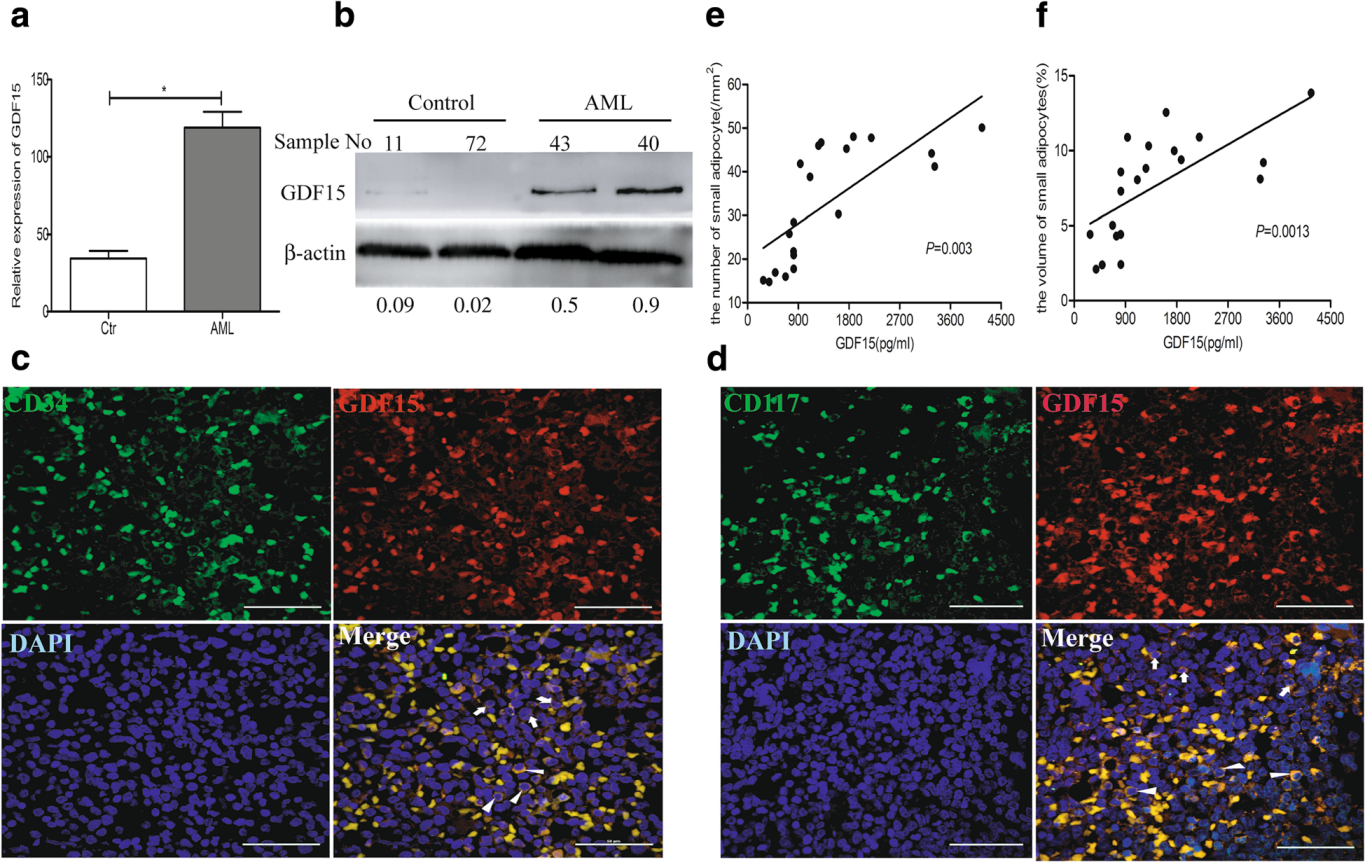
The relationship of GDF15 expression and small marrow adipocytes in AML patients. **a** RT-qPCR analysis of *GDF15* mRNA expression in BM from AML patients (*n* = 15) and the controls (*n* = 12). The results shown are from three independent experiments. *P < 0.05. **b** Western blotting analysis of GDF15 protein levels in BM from AML patients and the controls. The densitometry values of protein expression changes were indicated. β-actin protein was used as an internal control for Western blotting analysis. **c** and **d** Representative confocal images showed the expression of GDF15 and leukemic cell markers CD34 (**c**) or CD117 (**d**) in BM sections of AML patients. DAPI was used to stain the nuclei. White triangles showed the leukemic cells with GDF15+. White arrows showed the non-leukemic cells with GDF15. Scale bar represents 40 μm. **e** and **f** Scatter plot showed the positive correlation of small adipocyte volume (**e**) or small adipocyte number (**f**) with the level of GDF15 in BM of AML (*n* = 20, *R* = 0.6679, *P* = 0.0013, or *n* = 20, *R* = 0.7205, *P* = 0.003, Spearman correlation test)

To further investigate the contribution of GDF15 to small marrow adipocytes in AML patients, the expression of GDF15 was measured in the BM of AML patients. We collected the mononuclear cells from BM of the AML patients (*n* = 15) and controls (*n* = 12). Compared to the controls, primary AML blasts exhibited higher expression of GDF15 by RT-qPCR and Western blotting analyses (Fig. 5a-b). On the BM sections of AML patients, CD34^+^ or CD117^+^ cells, which were regarded as leukemic cells, were positive for GDF15 (Fig. 5c-d). However, part of GDF15^+^ cells exhibited no expression of CD34 or CD117, indicating that GDF15 can also be expressed from other cells besides primary AML blasts. Furthermore, we detected the level of GDF15 in BM aspirates and quantified both the number and the volume of small marrow adipocytes on the BM sections from the same AML patients. Linear analysis showed that the levels of GDF15 in BM aspirates were positively correlated with the number and the volume of small marrow adipocytes on the BM sections (Fig. 5e-f), suggesting that increased GDF15 was associated with small marrow adipocytes in AML.

## Discussion

In the present study, we investigated the possible mechanism on the generation of small adipocytes in AML patients by focusing on extracellular regulatory factors. We found that small marrow adipocytes were remodeled from larger marrow adipocytes in response to the release of GDF15 from leukemic cells. Accordingly, GDF15-induced small adipocytes could promote the proliferation of leukemic cells, indicating that GDF15 plays a critical role in the crosstalk between leukemic cells and adipocytes.

In this study, both on BM sections and in AML cell lines, our experiments showed that AML cells highly expressed GDF15. As a secretory protein, GDF15 might be released into the BM cavity by leukemic cells and have an effect on other cells in BM, including adipocytes. Certainly, non-leukemic cells also expressed GDF15 on BM sections (Fig. 5c-d). Our previous studies have demonstrated that GDF15 was highly expressed in leukemia-activated fibroblasts, suggesting that GDF15 was from many kinds of cells in BM of AML patients. Additionally, it has been reported that mature adipocytes can undergo morphologic changes, from mature adipocytes to small adipocytes (cancer-associated adipocytes) to fibroblast-like cells (adipocytes-derived fibroblasts) when they were activated by soluble factors derived from solid tumor cells [6]. Therefore, we guessed that GDF15 secreted by leukemic cells might play an important role in the conversion from adipocytes to leukemia-activated fibroblasts. Functionally, GDF15 was involved in the fibrosis of many organs [28–30]. Although both the fibroblasts and adipocytes can be differentiated from BMSCs, it is less known about the function of GDF15 in adipogenesis. However, it is well known that TGF-β1 and GDF15 are belonged to the TGF-β superfamily, which can prevent preadipocyte differentiation through cooperation with the Wnt signaling pathway [27]. Similarly, CTGF, a downstream mediator of TGF-β1 signaling in many cell types, has anti-adipogenic effects in primary adipocytes [31, 32]. It leads us to infer the possibility that GDF15 may play an important role in the marrow adipocyte differentiation of AML patients. Indeed, our results, acquired by exogenous addition rhGDF15 or neutralizing anti-GDF15 antibody, indicated that GDF15 can induce the transition of larger adipocytes into small adipocytes. This was further confirmed by the positive correlation between the levels of GDF15 and the number and volume of small adipocytes in AML patients. Meanwhile, our results were consistent with the findings reported by Chrysovergis K et al. In that study, they found that the transgenic mice treated with GDF15 expressing xenografts had smaller adipocytes compared to wild-type (WT) littermates [33].

Our results indicated that GDF15 could induce the adipocyte remodeling, that is the morphological transition of larger adipocytes into small adipocytes. It has been showed that the small adipocytes in mice highly expressed lipolytic genes [33]. Our results also showed that the expression of lipolytic genes including HSL and ATGL increased in the GDF15-induced small adipocytes, indicating that the marrow adipocytes remodeling might be dependent on lipolysis. However, much remains to be uncovered on the mechanism of lipolysis regulation by GDF15. It has been reported that GDF15 induced a significant ERK activation in the malignant progression of human cancer cell [34–36]. What’s more, ERK activation could cause HSL phosphorylation which leads to increased activity of the enzyme [37]. Thus, whether the increased HSL in GDF15 induced-small adipocytes is also dependent on ERK signal pathway requires further study. Adipose tissue is a plastic organ with the ability of a continuous remodeling, including of extension and regression depending on nutrient intake [38, 39]. The morphological, transcriptional and functional remodeling of adipocytes may be concurrent [39]. Indeed, we found that when the adipocytes were becoming small induced by GDF15, they had a stronger ability to promote the proliferation of leukemic cells (Fig. 4). Smaller adipocytes are frequently linked to higher metabolic activity since smaller adipocytes suggest greater utilization of fat storage for metabolism [40, 41]. Until now, the function of FFAs on the tumor cells was still controversial. On one hand, some reported that FFAs could inhibit the growth and progression of breast cancer cells [42, 43]. On the other hand, FFAs could promote the proliferation of leukemic cells and the metastasis of ovarian cancer cells [2, 44]. In our study, we found that with the high expression of lipolytic genes, the FFA levels increased during the process of small adipocyte formation. Since it has been reported that FFAs can be transported by FABP4 to AML cells [2], we considered that the leukemic cell rapid proliferation might rely on FFAs providing energy.

## Conclusions

In conclusion, leukemic cells mediated the marrow adipocyte remodeling from larger adipocytes to small adipocytes by secreting GDF15. In turn, small adipocytes enhanced the leukemic cell proliferation by providing FFA. From the point of bone marrow microenvironment, GDF15 might be a new target to treat the leukemia. Since our study was focused only on clinical samples, animal studies in the future would provide a better insight into the mechanism of GDF15 regulation on marrow adipocytes remodeling.

## Additional file

Additional file 1: **Figure S1.** The GDF15 expression in THP-1 cells with different treatment. (DOCX 172 kb)

## Abbreviations

AML: Acute myeloid leukemia
ATGL: Adipose triglyceride lipase
BM: Bone marrow
BMP: Bone morphogenetic protein
C/EBPα: CCAAT/ enhancer binding protein alpha
CM: Conditioned medium
CTGF: Connective tissue growth factor
DMEM: Dulbecco’s modified Eagle’s medium
FABP4: Fatty acid-binding protein 4
FBS: Fetal bovine serum
FFA: Free fat acid
GDF15: Growth differentiation factor-15
GDF6: Growth differentiation factor-6
HSL: Hormone-sensitive triglyceride lipase
MSC: Mesenchymal stem cells
ORO: Oil Red O
PPARγ: Peroxisome proliferator activated receptor gamma
RT-qPCR: Real-time quantitative PCR
