# Supplementary material for: Growth differentiation factor 15 contributes to marrow adipocyte remodeling in response to the growth of leukemic cells
Source: J Exp Clin Cancer Res. 2018 Mar 22;37:66. doi: 10.1186/s13046-018-0738-y (PMC5863796; doi:10.1186/s13046-018-0738-y)
Supplement: Supplementary file 1 — Figure S1. The GDF15 expression in THP-1 cells with different treatment. (DOCX 172 kb) [file 13046_2018_738_MOESM1_ESM.docx]

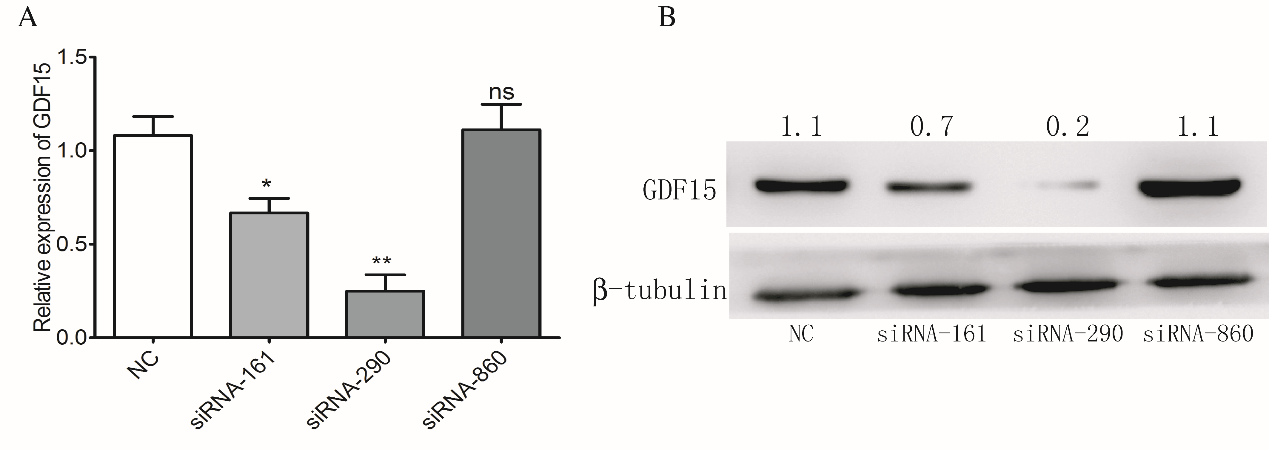


**Additional file 1: Figure S1. The GDF15 expression in THP-1 cells with different treatment.**

A and B. RT-qPCR (A) and Western blotting (B) analysis of GDF15 in THP-1 cells treated with three different GDF15 siRNA (siRNA-161, 290, 860) or negative control (NC). The densitometry values of protein expression changes were indicated. β-tubulin was used as an internal control for RT-qPCR and Western blotting analysis.* P<0.05, **P<0.01.
